# Genome-wide screens reveal *Escherichia coli* genes required for growth of T1-like phage LL5 and V5-like phage LL12

**DOI:** 10.1038/s41598-020-64981-7

**Published:** 2020-05-15

**Authors:** Denish Piya, Lauren Lessor, Brian Koehler, Ashley Stonecipher, Jesse Cahill, Jason J. Gill

**Affiliations:** 1 Department of Biochemistry & Biophysics, Texas A&M University, College Station, TX 77843 USA; 2 Center for Phage Technology, Texas A&M University, College Station, TX 77843 USA; 3 Department of Animal Science, Texas A&M University, College Station, TX 77843 USA; 4 Present Address: Department of Bioengineering, University of California Berkeley, Berkeley, CA 94704 USA

**Keywords:** Microbiology, Molecular biology

## Abstract

The host factor requirements of phages and mechanisms of mutational phage insensitivity must be characterized for rational design of phage cocktails. To characterize host dependencies of two novel *Escherichia coli* phages, the T1-like siphophage LL5 and the V5-like myophage LL12, forward genetic screens were conducted against the Keio collection, a library of single non-essential gene deletions in *E. coli* str. BW25113. These screens and subsequent experiments identified genes required by phages LL5 and LL12. *E. coli* mutants deficient in heptose II and the phosphoryl substituent of heptose I of the inner core lipopolysaccharide (LPS) were unable to propagate phage LL5, as were mutants deficient in the outer membrane protein TolC. Mutants lacking glucose I of the LPS outer core failed to propagate LL12. Two additional genes encoding cytoplasmic chaperones, PpiB and SecB, were found to be required for efficient propagation of phage LL5, but not LL12. This screening approach may be useful for identifying host factors dependencies of phages, which would provide valuable information for their potential use as therapeutics and for phage engineering.

## Introduction

*Escherichia coli* is a Gram-negative facultative anaerobic bacterium which is commonly found as a member of the commensal gut flora in mammals^1^. While commensal *E. coli* strains generally do not cause disease in humans, multiple strains of *E. coli* have acquired virulence factors and may cause disease with symptoms ranging from mild discomfort to life-threatening bacteremia. These strains have been categorized into several pathotypes, including enteropathogenic *E. coli* (EPEC), enterohemorrhagic *E. coli* (EHEC), enterotoxigenic *E. coli* (ETEC), enteroaggregative *E. coli* (EAEC), enteroinvasive *E. coli* (EIEC), and diffusely adherent *E. coli* (DAEC)^2^.

Enterotoxigenic *E. coli* strains can be distinguished from other *E. coli* pathotypes by the presence of heat-labile enterotoxin (LT) and/or heat-stable enterotoxin (HT)^2^. These enterotoxins induce traveler’s diarrhea (TD), characterized by mild to severe watery diarrhea^2^, which may be accompanied by nausea, vomiting, abdominal pain, fever or blood in stool^3^. TD is one of the most common illnesses contracted by people from developed countries during international travel, with ETEC being the major causative agent of TD in Latin America, Africa, South Asia and the Middle East^4^. TD typically self-resolves or may be successfully treated with antibiotics, but the global increase in the emergence of antibiotic resistance warrants evaluation of alternative treatment approaches^4^.

Bacteriophages (phages) are the natural viral predators of bacteria. Due to the ongoing emergence of multidrug-resistant bacteria and limited development of new antibiotics, there has been a renewed interest in the use of phages as antimicrobials^5–9^. However, knowledge of the basic biology of phages outside of a number of well-studied model organisms is limited. Phages are highly diverse, and they are constantly co-evolving with their bacterial hosts^10,11^. In the use of phages as therapeutics, many novel phages need to be isolated and deployed^12^; for phage therapy to succeed, a thorough understanding of phage-host interactions on a broader scale is required^5^.

Since phages must intimately interact with their hosts to propagate, bacteria can develop resistance against phages by mutational loss of even a single gene. Rational formulation of phage cocktails includes the selection of phages with genetically distinct mechanisms of mutational resistance, which can delay the emergence of bacterial resistance to the phage cocktail as a whole. With the availability of modern genetic resources, host-phage interactions can be studied more efficiently on a larger scale. A number of genome-wide screens have been conducted to study host factors required for viral replication in organisms such as HIV^13^, Influenza virus^14^, phages λ^15^, T7^16^, P22^17^ and HK97^18^. These screens can also be optimized specifically for identification of cell surface host factors that may serve as phage receptors. We conducted forward-genetics screens of the *E. coli* phages LL5 and LL12 against the Keio collection, a library of single-gene deletions of all non-essential genes in *E. coli* K-12 strain BW25113^19^, in order to characterize major host functions required for propagation of these two potentially therapeutic phages. Identification of host dependencies allows for the rational design of phage cocktails and opens avenues for phage engineering to overcome host dependencies. The results of this screen are discussed in terms of host factors required for infection and propagation of phages LL5 and LL12.

## Results and Discussion

### Characterization of phages LL5 and LL12

**Figure 1:**
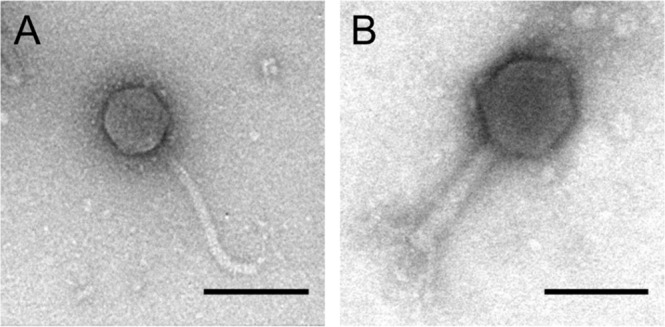
Transmission electron micrographs of phages LL5 (**A**) and LL12 (**B**). Phage LL5 has a capsid diameter of 61 nm (±2 nm) and a flexible, non-contractile tail 156 nm (±10 nm) in length. Phage LL12 has a capsid diameter of 86 nm (±2 nm) and a non-contractile tail 112 nm (±4 nm) in length. Dimensions are an average of ten measurements and the error represents standard deviation. The scale bar denotes 100 nm.

Phages LL5^20^ and LL12^21^ were isolated and their genomes sequenced as described previously. Phage LL5 is a T1-like siphophage with >90% identity at the DNA level to phage TLS, and phage LL12 is a V5-like myophage which is most closely related to phage rV5 but is also more distantly related to phage phi92. Transmission electron microscopy showed that phage LL12 is a myophage with a head diameter of ~85 nm and a tail of ~110 nm in length with a pronounced baseplate, while LL5 is a siphophage with a head diameter of ~60 nm and a flexible tail ~150 nm in length (Fig. 1).

Infection by STEC strains can result in watery or bloody diarrhea, hemolytic uremic syndrome, microangiopathic hemolytic anemia and thrombocytopenia^22^. *E. coli* strains belonging to several pathotypes tend to be clonal and are grouped as serotypes based on the O-antigens (lipopolysaccharide) and H-antigens (flagella)^2^. As phage LL12 bears similarity to phages rV5 and ΦAPCEc02, both of which infect STEC serotype O157:H7^23,24^, we sought to determine if phages LL5 and LL12 are also able to infect STEC representatives.

Phages LL5 and LL12 were spotted on soft agar overlays of STEC strains and their efficiency of plating (EOP) compared to the Keio parental strain BW25113. Phage LL5 was unable to form plaques on any of the tested STEC strains, and phage LL12 exhibited EOPs of close to 1 on STEC strains of serotypes O157:H7, O145:NM, O121:H19, O146 and O121:H19, demonstrating a relatively broad host range among STEC serotypes (Table 1).

**Table 1:** Host range of phages LL5 and LL12.

STEC serotype	Isolate ID	Phage LL5 EOP	Phage LL12 EOP	LPS Core types^b^
Not STEC	BW25113	1.0	1.0	K-12
O157:H7^a^	USDA-FSIS 380–94	—	0.8	R3
O104:H21	ATCC BAA-178	—	—	
O145:NM^a^	83–75	—	0.7	R1, K-12
O26:H11^a^	H30	—	—	R3
O111:H-^a^	JBI-95	—	—	R3
O121:H19	ATCC BAA-2219	—	0.7	
O146	ATCC BAA-2217	—	1.0	
O103:H11	ATCC BAA-2215	—	—	
O145:Nonmotile	ATCC BAA-2192	—	—	R1, K-12
O26:H11	ATCC BAA-2196	—	—	R3
O45:H2	ATCC BAA-2193	—	—	
O103:H2^a^	CDC 90–3128	—	—	R3
O121:H19^a^	CDC 97–3068	—	0.6	
O45:H2^a^	CDC 96–3285	—	—	

^a^Sources of these isolates are described in^56^.

^b^LPS core types information obtained from^42^.

Phage LL5 and LL12 were tested for their ability to infect Shiga toxin-producing *Escherichia coli* (STEC) by spotting serially diluted phages on the soft agar lawns of respective STEC isolates. The efficiency of plating (EOP) is relative to the number of plaques formed on the Keio collection parental *E. coli* strain BW25113. Cells marked with “-” indicate an EOP of less than 10^−7^ (insensitive to phage). The data is the average of two biological replicates.

### Development and optimization of screening assay

Multiplicity of Infection (MOI) is the ratio of the number of the phages to host cells in a culture. The purpose of the screen was to identify host genes required for the phage to successfully infect the cell and produce progeny, as measured by the phage’s ability to suppress bacterial growth in liquid culture. To determine this, it was imperative to optimize MOI for each phage as excessively high MOI’s could result in bacterial growth inhibition even if the phage were able to infect the cells but still not produce progeny, while MOI’s which were too low could result in false positive results^25^. Initially, the lowest input phage concentration required to control growth of parental BW25113 in liquid culture after 8 hr incubation at 37 °C was determined. A tenfold higher phage concentration was applied in this screen so as to minimize false positives. The number of bacterial cells inoculated by the 96-pin replicator were determined by viable counts. Based upon the cells inoculated and PFU of phages used, the initial MOI of LL5 and LL12 used in this screen was 1.0 and 0.001, respectively. Following this screen, only mutants that were also associated with significant plating defects on lawns of the mutant strains (EOP reduced by ~20-fold or more compared to plating on the BW25113 parent) were retained for further study (Tables S1 and S2).

### Genes required for propagation of phage LL5

Phage LL5 was screened against the 3,985 single-gene knockouts of the Keio collection as described in the Materials and Methods and in the supplementary text. The OD values of the strains observed during the screen in the absence and presence of LL5 are reported in Table S3. Following screening, confirmation of the defect by plating and *in trans* complementation of each mutation, eight genes were determined to be required for efficient propagation of phage LL5 (Table 2). Strains deleted for genes *gmhA*, *waaE*, *waaC*, *waaP*, *waaF* and *tolC* showed severe plating defects with the EOP of phage LL5 less than ~10^−7^. This plating defect was also observed in the P1-transduced *waaP* mutant. The plating efficiency of phage LL5 could be restored when the respective genes were provided *in trans* (Table 2). Two additional Keio mutants, *secB* and *ppiB*, exhibited milder defects in supporting phage LL5 growth, with EOP reductions of ~10- to 100-fold relative to the parental *E. coli* strain BW25113; these mutants could be transduced by P1 into the parental background and could also be complemented *in trans* (Table 2).

**Table 2:** *E. coli* genes required for efficient infection by phages LL5 and LL12.

Phage	Gene	Detection method	EOP	Complemented EOP
LL5	*gmhA*	Targeted screen	<7.5 × 10^−8^	0.8 ± 0.2
	*waaE*	Targeted screen	<7.5 × 10^−8^	1.0 ± 0.5
	*waaC*	Targeted screen	<5.3 × 10^−7^	0.7 ± 0.3
	*waaP*^*#*^	Initial screen	<7.5 × 10^−8^	2.2 ± 2.0
	*waaF*	Targeted screen	<7.9 × 10^−7^	0.7 ± 0.3
	*tolC*	Targeted screen	<5.3 × 10^−7^	0.4 ± 0.3
	*secB*^*#*^	Initial screen	0.06 ± 0.02	0.2 ± 0.04
	*ppiB*^*#*^	Initial screen	0.09 ± 0.05	1.5 ± 0.3
LL12	*gmhA*	Initial screen	<4.4 × 10^−9^	1.1 ± 0.4
	*waaE*	Targeted screen	<4.4 × 10^−9^	1.1 ± 0.5
	*waaC*	Targeted screen	<5.1 × 10^−9^	0.9 ± 0.4
	*waaP*^*#*^	Initial screen	0.02 ± 0.01	1.0 ± 0.4
	*waaF*	Targeted screen	<6.5 × 10^−9^	1.5 ± 0.5
	*waaG*	Initial screen	5.1 × 10^−6^ ± 1.0 × 10^−6^	1.1 ± 0.2

The genes required for phage infection cycle can be determined by testing the efficiency of plating. Eight genes were found to be required for phage LL5 plaque formation, whereas six genes were required for phage LL12. The kanamycin resistance cassette in the Keio strains were P1 transduced into parental BW25113 when possible in the initial screen, as denoted by “#”. The plating phenotype was complemented in P1 transductants, when applicable. The data represents average and standard deviation of three biological replicates. The OD values for the gene “hits” obtained in the initial screen are provided in the supplementary information Table S3.

**Figure 2:**
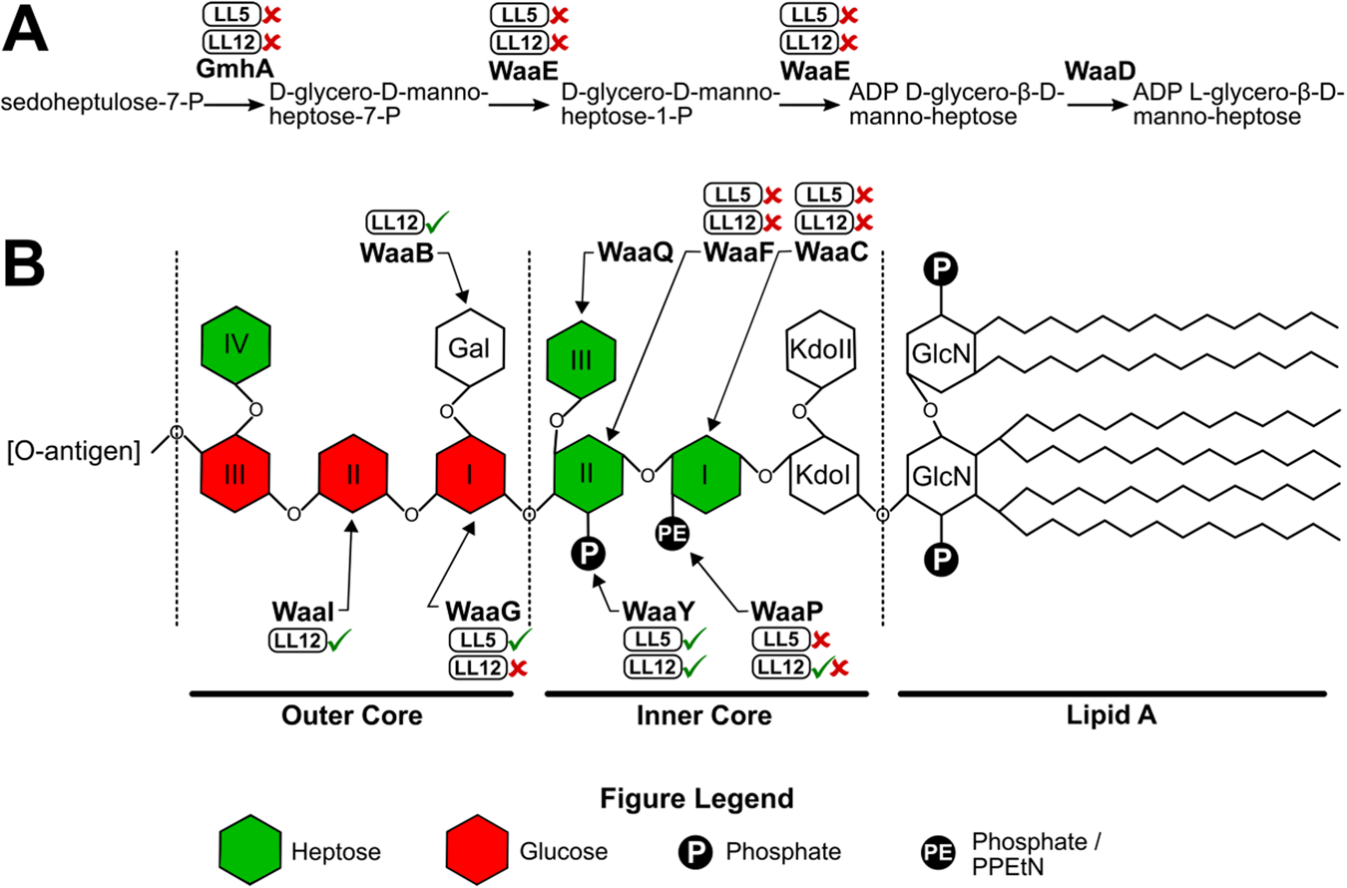
Genes and biosynthetic pathway of the *E. coli* core lipopolysaccharide (LPS) required for replication of phages LL5 and LL12. Proteins in the pathway are denoted in bold and label each step in biosynthesis. The ability of phages LL5 and LL12 to form plaques on mutants deficient at each step of the pathway are denoted by a green checkmark (forms plaques) or red “X” (does not form plaques) as denoted above each protein name. Panel A: The nucleotide sugar precursor ADP L-glycero-β-D-manno-heptose is used as a substrate for the transfer of heptose (green in panel B) to the *E. coli* core LPS. ADP L-glycero-β-D-manno-heptose is synthesized from sedoheptulose-7-P via a pathway comprised of *gmhA*, *waaE* and *waaD*. Both *gmhA* and *waaE* are required for growth of phages LL5 and LL12. Panel B: LPS is composed of four distinct domains: Lipid A, inner core, outer core and O-antigen. The enzymes responsible for the addition of sugar residues and phosphoryl constituents relevant to this study are denoted. WaaC, WaaF, WaaQ, WaaG, WaaI and WaaB add hexo or hepto sugar residues to LPS, and WaaP and WaaY add phosphoryl substituents to heptose residues I and II, respectively. WaaC, WaaP and WaaF are required for plaque formation by phage LL5. WaaC, WaaF and WaaG are required by LL12, and absence of WaaP (checkmark and “X”) results in a ~50-fold reduction in plating efficiency for phage LL12. Panel A is adapted from^54^ and panel B from^55^.

The genes *gmhA*, *waaE*, *waaC*, *waaP* and *waaF* are parts of the core LPS biosynthesis pathway, and the severe plating defects associated with multiple genes in this pathway indicate that phage LL5 requires an intact core LPS for infection. WaaP adds phosphate or 2-aminoethyl diphosphate (PPEtN) to Hep I of the inner core LPS (Fig. 2B)^26^, and defects in *waaP* or earlier steps cause the “deep rough” phenotype associated with significant outer membrane defects, including increased detergent sensitivity, aberrant LPS distribution and greatly reduced OMP incorporation^27^. Addition of Hep II by WaaF is the last step in the LPS pathway with a strong observed plating defect for LL5, suggesting that the Hep II residue of the core LPS is also required for infection by phage LL5. The plating efficiency on the *waaY* mutant was normal and only a mild defect was observed on the *waaQ* mutant (Table S1), indicating that these genes are not required for LL5 infection.

The LL5 predicted central tail protein gp51 is 98% identical to the TspJ tail protein of the T1-like phage TLS (TLS gp51, YP_001285540). Approximately 3 kb downstream from gp51, LL5 gp57 is similar to other putative T1-like tail fibers only in its N-terminal domain, with the C-terminal domain more closely related to putative tail fiber proteins found in T5-like phages such as DT57C and DT571/2^28^. The residues 273–605 of LL5 gp57 is 57% identical with a C-proximal region spanning residues 515 to 830 of the 1,076-residue DT57C LtfA protein (YP_009149889), which is within the host specificity region of this protein^29^. Phage TLS utilizes the outer membrane protein TolC and the LPS core to recognize and infect its *E. coli* host^30^. Since the inner core LPS requirement of phage LL5 appears similar to that of phage TLS^30^, the dependence of LL5 on TolC for infection was also tested. The plating efficiency of phage LL5 in a *tolC* deletion mutant was less than 5 × 10^−7^, and this phenotype could be complemented *in trans* (Table 2). While the data presented here indicates phage LL5 requires an intact LPS inner core and the TolC OMP for infection, it should be noted that TolC is also required for normal outer membrane assembly and these mutants also show the “deep rough” phenotype^31^, and thus it is difficult to disentangle the effects of deletions in *tolC* and genes involved in LPS inner core synthesis in phage LL5 infection. Moreover, it has been previously reported that *tolC* mutants are also resistant to LPS specific phages^32^, so it is unclear if phage LL5 interacts directly with TolC for successful infection, or the changes in the LPS structure due to loss of TolC confers LL5 insensitivity. It is also not clear which of LL5’s two putative tail fibers, the central fiber gp51 and L-shaped side fiber gp57, recognize which surface features.

Apart from the genes encoding surface features, the plating efficiency of phage LL5 was also found to be reduced in strains with deletions in two other genes, *secB* and *ppiB* (Table 2), which are chaperones that contribute to protein translocation and proline peptide bond isomerization, respectively^33,34^. SecB, a tetrameric cytoplasmic chaperone, is a component of the general secretory (Sec) system that transports proteins synthesized in the cytoplasm, post-translationally, to extracytoplasmic compartments. Post-translational transport is primarily preferred for periplasmic and outer membrane proteins^35,36^. Eighteen *E. coli* proteins have been reported to be dependent on SecB-mediated translocation^33,36^. We do not know if any of these SecB-dependent proteins play a role in the infection cycle of phage LL5, or if possibly the accumulation of cytoplasmic protein aggregates in *secB* mutants hampers phage replication. In the absence of SecB, other cytoplasmic chaperones have been reported to be upregulated to stabilize secretory proteins during their delayed translocation and/or to rescue protein aggregates^37^. This compensatory mechanism by other chaperones may be the reason why the EOP defect of phage LL5 in the *secB* mutant is relatively mild (EOP = 0.06).

PpiB, which belongs to peptidyl-prolyl *cis/trans* isomerase (PPIase) superfamily of proteins, catalyzes protein folding at the peptide bonds preceding proline residues^34^. Although PPIases play a role in several biological processes, there is no evidence of any biological process depending solely on any PPIases^34^. The genome of *E. coli* K-12 encodes eight PPIases, belonging to three families: FKPBs, cyclophilins and parvulins^34^. The cyclophilins family consist of PpiA and PpiB, which are periplasmic and cytoplasmic proteins, respectively^34^. To our knowledge, there are two other reported instances of the requirement of PPIases for phage infection. SlyD, belonging to FKBP family of PPIases, has been shown to be required for plaque formation by the ssDNA phage ΦX174^38^ by stabilizing the phage lysis protein E^39^. FkpA, a periplasmic PPIase, is required for infection by phage HK97 and some related coliphages, and appears to be required to support phage DNA entry into the cell^18^. Since the infection cycle of phage LL5 has not been characterized, it is difficult to explain which aspect of phage replication is affected by the absence of PpiB.

### Genes required for propagation of phage LL12

Phage LL12 was screened against the 3,985 single-gene knockouts of the Keio collection as described in the Materials and Methods and in the supplementary text. The OD values of the strains observed during the screen in the absence and presence of LL12 are reported in Table S3. Following confirmation of the plating defect and genetic complementation, six genes were determined to be required for efficient propagation of phage LL12 (Table 2). All mutants in which phage LL12 showed plating defects were deleted for genes in the LPS biosynthesis pathway. Phage LL12 showed severe plating defects (EOP < 10^−8^) in *gmhA*, *waaE*, *waaC* and *waaF* deletions, and an EOP of ~10^−6^ in the *waaG* deletion. Deletion of *waaP* resulted in a milder plating defect (EOP ~0.02). These defects could be restored when the respective genes were supplied *in trans* (Table 2).

The functions of genes *gmhA*, *waaE*, *waaC*, *waaP* and *waaF* in LPS biosynthesis have been explained in context of phage LL5 above. WaaG links glucose (Glc) I to Hep II of the LPS inner core (Fig. 2B)^40,41^, and marks the start of the outer core domain of the *E. coli* LPS. Sugar residues Glc II and galactose (Gal) are linked to Glc I by WaaI and WaaB respectively^27^. The plating efficiency of phage LL12 in the respective *waaI* and *waaB* mutants were close to wild type (~0.5) suggesting that Glc II and the Gal sidechain do not play significant roles in phage LL12 infection (Table S2). The strongly reduced EOP of phage LL12 on *waaG* deletions suggests a crucial role of the outer core Glc I in the host recognition mechanism of phage LL12. A milder plating defect was observed in the *waaP* mutant (Table 2), which lacks the phosphate/PPEtN modification to Hep I, the phosphate modification to Hep II by WaaY, and the addition of Hep III by WaaQ^26^. However, the *waaQ* deletion did not appear in the initial screen, and deletion of *waaY* shows only a minor reduction in plating efficiency (EOP ~0.1), suggesting that LL12 may also require the phosphate/PPEtN modification to Hep I for successful infection.

The Hep II - Glc I linkage is conserved in K-12, and R1 - R4 LPS core types in *E. coli*^42^. As shown in Table 1, phage LL12 is able to infect *E. coli* strains with K-12, R1 and R3 LPS core types, which is consistent with the finding that the Gal sidechain residue linked to Glc I in the K-12 core and the residues downstream of Glc I are not required for efficient phage infection. To our knowledge, LL12 is the first V5-like phage for which host factor requirements have been characterized.

Like the related phages rV5, phi92 and ΦAPCEc02, phage LL12 encodes an extensive set of predicted tail fibers: gp27, gp29, gp32, gp33, gp36, gp41 and gp42^42^. All seven of these LL12 putative tail fibers are similar to putative tail fibers found in rV5 and ΦAPCEc02, with protein identities (by Dice coefficient) ranging from 4–100% (Table S4). Six of these seven proteins are also detectable in the more distantly-related phage phi92, with three of these, gp29, gp33 and gp42 producing alignments to nearly the full-length phi92 proteins 147, 142 and 141, respectively (Table S4). CryoEM reconstructions of phi92 have indicated that this phage possesses multiple sets of tail fibers that are mounted to the baseplate in downward, sideward, and upward orientations^43^. These multiple tail fibers may contribute to a broadened host range in this phage and its relatives^43^. The electron density of the downward-facing tail fiber was assigned to gp143, which is not conserved in LL12^42,43^. LL12 gp27 shows weak similarity to the N-terminus of phi92 gp150, which is predicted to form downward-facing tail spikes in cryoEM reconstructions^43^. LL12 gp41 also possesses similarity to rV5 gp41 (Table S4), however LL12 gp41 is missing the C-terminal chaperone of endosialidase domain (pfam13884) of rV5 gp41 spanning residues 1151–1200. Based upon the sequence similarities of their tail fibers (Table S4), other closely related V5-like phages such as rV5, ΦAPCEc02, and the O157:H7 typing phages 4, 5 and 14 are likely to have similar requirements to initiate infection as phage LL12.

Genetic analysis has established phage LL12 requires an intact *E. coli* LPS outer core for successful propagation in a K-12 background, which lacks an intact O-antigen. However, LL12 is also able to infect multiple different serotypes of *E. coli* with various full-length O-antigens (Table 1). In the case of phage P1, which recognizes the LPS outer core^44^, the extensive O-antigen expressed by hosts such as *E. coli* O157:H7 and *Salmonella* Typhimurium is able to obscure the LPS core. Loss of the O-antigen in these strains results in bacterial sensitivity to phage P1^45,46^. This observation suggests that phage LL12 has developed a mechanism to deal with the presence of O-antigen that may mask its receptor in the LPS core. Several phages are known to have evolved mechanisms to penetrate bacterial O-antigen in order to reach their major receptors on the cell surface. The tail spike protein (TSP) of *Salmonella* phage P22 recognizes O-antigen as its receptor and also has endorhamnosidase activity and cleaves its glycosidic linkages resulting in the shortening of the O-antigen^47,48^. Coliphage G7C also expresses tail spikes with enzymatic activity against O-antigen that is involved in phage adsorption^49^. Absence of any such enzymatic domains in the putative tail proteins of phage LL12 suggest that LL12 might have other mechanisms to overcome the bacterial O-antigen barrier.

## Conclusions

Phages LL5 and LL12 were initially isolated against pathogenic *E. coli* hosts and phage LL12 was shown to infect representatives of several prominent STEC serovars. Phage LL5 is closely related to phage TLS and encodes two putative receptor binding proteins: a central tail spike (gp51) and tail fiber (gp57), the latter of which is closely related to the L-shaped fibers of coliphage T5. LL12 is a large (~136 kb) myophage and a member of the V5-like phages, which are known to infect pathogenic *E. coli* strains. A total of eight and six *E. coli* genes were found to severely affect the propagation of phages LL5 and LL12, respectively (Table 2). Phage LL5 exhibited severe plating defects (<10^−7^) in *E. coli tolC* and other mutants defective in LPS inner core biosynthesis, which suggest that LL5 requires the outer membrane protein TolC, and the Hep II and phosphoryl modification of Hep I of the LPS inner core, to infect its host. Phage LL5 also exhibited mild plating defects in *E. coli* mutants defective in *ppiB* and *secB*, which are cytoplasmic chaperones that may be involved in the production of phage components or proteins required for proper receptor expression. Phage LL12 showed severe plating defects in *E. coli* mutants defective for LPS inner and outer core synthesis, indicating that LL12 requires the LPS outer core Glc I for infection.

The number of host genes required for phage propagation detected by the liquid culture-based method used in this study is similar to those found by similar screens. In one of the earliest screens of this kind, Qimron *et al*.^16^ screened the entire Keio collection against the virulent coliphage T7 by a replica-plating method and identified 11 genes required for T7 infection; nine of these encoded LPS biosynthetic functions, and LPS is the T7 receptor. By assessing plaque formation on soft agar overlays, Cumby *et al*.^18^ screened an 815-member subset of the Keio collection against the lambdoid phage HK97 and identified three genes required for phage infection, one of which was the phage receptor LamB. Bohm *et al*.^17^ used a highly sensitive approach to screening *Salmonella* phage P22 against a saturating transposon insertion library, and identified some 312 genes with some effect on phage infection as measured by the persistence of phage DNA in culture. However, many of these mutants were still able to support plaque formation and the strongest phenotypes tended to be observed in mutants with defects in the production of LPS, which also serves as the P22 receptor.

This study was initiated with the screening of phages LL5 and LL12 against the Keio library to investigate the host factors required for robust phage propagation. Initial screens identified 37 *E. coli* genes necessary for phage LL5 and LL12 propagation (Tables S1, S2 and supplementary text), but on further analysis only five of these were found to be associated with significant plating defects, and an additional four genes were identified by targeted re-testing of specific gene knockouts. These observations highlight the generally noisy nature of high-throughput screens and the requirement for additional confirmatory experiments following screening.

## Methods

### Bacterial strains and plasmids

The Keio collection was purchased from Thermo Scientific^19^. Keio strains deleted for *tolC* and *waaC* were obtained directly from the Coli Genetic Stock Center at Yale University. The Keio strain deleted for *waaF* was purchased from Dharmacon, Inc. Strains from the ASKA library used for complementation were purchased from National BioResource Project (NIG, Japan)^50^. To complement phenotypes associated with *tolC*, *waaC* and *waaF*, respective genes from *E. coli* str. MG1655 were cloned into the pBAD24 vector and expressed *in trans* as previously described^51^. The parental *E. coli* strain BW25113 was obtained from Ry Young (Texas A&M University, College Station, TX). *E. coli* strains from the Keio collection and their transductants were cultured in LB (Lennox) broth [10 g L^−1^ Bacto tryptone (BD), 5 g L^−1^ Bacto yeast extract (BD), 5 g L^−1^ NaCl (Avantor)] or LB agar [LB broth amended with 15 g L^−1^ Bacto agar (BD)] at 37 °C amended with 30 μg mL^−1^ kanamycin (LB kan) and strains containing plasmids from the ASKA library were maintained on LB amended with 10 μg mL^−1^ chloramphenicol (LB cm). Plasmid DNA from ASKA library strains was extracted using a QIAprep Spin Miniprep Kit (Qiagen). In complementation experiments with the ASKA plasmids, LB plates or top agar were supplemented with 0.05–0.1 mM IPTG to induce protein expression^50^. Strains containing pBAD24-based plasmids were maintained on LB amended with 100 μg mL^−1^ ampicillin (LB amp). In complementation experiments with pBAD24-based plasmids, LB plates were supplemented with 0.1 mM L-arabinose. L-arabinose was omitted during complementation of *tolC* because of the toxicity of TolC overexpression. Leaky expression of TolC from the uninduced complementing plasmid was sufficient for restoring the plating efficiency of phage LL5. All primers used in this study will be provided upon request.

### Phage isolation and culture

The phages LL5^20^ and LL12^42^ were isolated against clinical isolates of enterotoxigenic *E. coli* (ETEC) obtained from John Deaton (Deerland Enzymes, Kennesaw, GA) as described previously. Both phages were subsequently cultured using *E. coli* strain DH5α as host. Phage lysates were prepared by the confluent plate lysis method^52^ using LB (Miller) bottom plates (10 g L^−1^ Bacto tryptone, 5 g L^−1^ Bacto yeast extract, 10 g L^−1^ NaCl, 15 g L^−1^ Bacto agar) and top agar consisting of 10 g L^−1^ tryptone, 10 g L^−1^ NaCl, 5 g L^−1^ Bacto agar. Phages were harvested and stored as filter-sterilized (0.22 µm) lysates in lambda diluent (25 mM Tris-HCl pH 7.5, 100 mM NaCl, 8 mM MgSO_4_, 0.01% w/v gelatin) at 4 °C.

Plaque assays were conducted using both spot titer and full-plate titration methods^52^. For spot titers, 10 µL of serially diluted phage was spotted on solidified lawns of 4 ml top agar inoculated with 100 μL of a fresh overnight host culture prepared as described above. For full-plate titers, 100 μL of serially diluted phage was mixed with 100 μL of host culture in 4 ml of molten top agar and poured over LB plates as described above. Plaques were enumerated after 16–18 h incubation at 37 °C. The efficiency of plating (EOP) was calculated as the ratio of the number of plaques appearing on the lawn of a test strain to the number of plaques on the reference strain.

### Screening and confirmation of phage-insensitive mutants

The goal of this screen was to identify genes whose deletion produced major defects in the ability of the phage to replicate. This was determined by the ability of the phage to suppress growth in liquid culture of each mutant strain to the same extent as the fully sensitive, parental host. In order to optimize the input phage concentrations and incubation times, stocks of phages LL5 and LL12 were serially diluted in fresh LB and 160 µL of each dilution was aliquoted into 96-well sterile transparent polystyrene flat-bottom plates (Greiner Bio-one). The plates were then inoculated with the Keio parental strain BW25115 using a 96-pin replicator (Phenix) and incubated at 37 °C for 6, 8, 10 and 18 hrs. The optical density (OD) at 550 nm was measured in a Tecan M200 plate reader at each time interval and the average OD was analyzed to determine the lowest phage concentration that inhibited bacterial growth. After determining the lowest concentration of phage LL5 or LL12 that inhibited the growth of parental BW25113 strain, a ten-fold higher concentration of phages was used in the high-throughput screen to minimize false positives.

The Keio collection consists of 90, 96-well plates containing two independently-generated sets of 3,985 single-gene knockouts in the *E. coli* BW25113 background^19^. The Keio strains were replicated into 96 well sterile polypropylene U-bottom microplates (Greiner Bio-one) containing LB kan + 8% glycerol using sterile plastic 96-pin replicators (Phenix). Plates were incubated at 37 °C overnight and stored frozen at −80 °C. These plates were used as the working stocks for the following screens. The odd- and even-numbered plates have identical gene deletion mutants created by independent experiments^19^, and only the odd-numbered 45 plates were used for the initial screen. Initial screens were conducted in 96-well sterile transparent polystyrene flat-bottom plates (Greiner Bio-one). Phages LL5 and LL12 were diluted in fresh LB to obtain working stocks of 10^6^ PFU mL^−1^ for LL5 and 10^3^ PFU mL^−1^ for LL12. 160 µL of the phage working stocks were aliquoted into all wells, Keio strains were inoculated into the phage lysates from the 96-well working stocks with 96-pin replicators, and the plates were incubated for 8 hours at 37 °C. The OD_550_ was measured and the wells with OD_550_ higher than the predetermined cutoff values (0.2 for phage LL5 and 0.11 for phage LL12) were scored as positive for growth.

The positive mutants obtained from the first screen were verified by repeating the assay with the same strains and their corresponding mutant strains from the even-numbered Keio collection plates, side-by-side with eight replicates per assay. Mutants that returned mean OD_550_ above the designated cutoff in either the even- or odd-numbered set were retained for further characterization by measurement of phage efficiency of plating (EOP) by spot assays on soft agar lawns^52^ as described above. EOP was calculated as the number of plaques observed on the mutant strain divided by the number of plaques observed on the parental *E. coli* strain BW25113. Mutants with EOP’s of less than ~0.05 were confirmed by enumerating plaques on full plates. When possible, mutant alleles were moved into the parental BW25113 background by P1 transduction using the kanamycin resistance cassette as the selectable marker^53^. All gene disruptions were confirmed by PCR using primers flanking the predicted insert followed by sequencing of the PCR product to confirm disruption of the gene. All mutants were complemented by transforming the original Keio mutant or its P1 transductant with a plasmid expressing the corresponding gene.

## Supplementary information

Supplementary Materials.

## Data Availability

All data generated in this study are included in the main text or supplementary information.
