# Supplementary material for: Genome-wide screens reveal Escherichia coli genes required for growth of T1-like phage LL5 and V5-like phage LL12
Source: Sci Rep. 2020 May 15;10:8058. doi: 10.1038/s41598-020-64981-7 (PMC7229145; doi:10.1038/s41598-020-64981-7)
Supplement: Supplementary file 1 — Supplementary Materials. [file 41598_2020_64981_MOESM1_ESM.pdf]

Supplementary materials

Genome-wide screens reveal *Escherichia coli* genes required for growth of T1-like phage LL5 and rV5-like phage LL12

Denish Piya [1,2,†], Lauren Lessor [2], Brian Koehler [2], Ashley Stonecipher [2], Jesse Cahill [1,2], Jason J. Gill [2,3,*]

[1] Department of Biochemistry & Biophysics, Texas A&M University, College Station, TX 77843, USA
[2] Center for Phage Technology, Texas A&M University, College Station, TX 77843, USA
[3] Department of Animal Science, Texas A&M University, College Station, TX 77843, USA

* Correspondence: jason.gill@tamu.edu (J.J.G); Tel.: +1-979-458-9286
† Currently at Department of Bioengineering, University of California Berkeley, Berkeley, CA 94704, USA

Determination of genes required for phage propagation

The Keio collection consists of a total of 3,985 individual gene knockout mutants in the *E. coli* K-12 strain BW25113. Each gene knockout is represented twice in the collection (the results of two independent experiments) [1], thus the total collection contains 7,970 mutants, with each independent gene knockout mutants represented with even and odd numbers. Phages LL5 and LL12 were screened against the entire odd-numbered series of 3,985 Keio single-gene knockouts as described in Materials and Methods. *E. coli* mutants that were unable to support phage growth, as indicated by their growth to an OD_{550} of at least 0.2 or 0.11 at 8 h in the presence of phage LL5 or LL12, respectively, were considered positive hits in this initial screen. Using this selection criteria, 37 knockout mutants (21 mutants for each phage) were selected for further investigation (Tables S1, S2). For each of these initial hits, the screening experiment was repeated using the same odd-numbered mutant and its even-numbered counterpart from the collection. From this second experiment, 11/21 mutants identified against LL5 and 9/21 mutants identified against LL12 were found to produce the same phenotype in at least one of the paired knockouts, and these were retained for further study.

The efficiency of plating (EOP) of phage LL5 on the retained mutant strains was determined by spot titer. The observed plating efficiency of phage LL5 was reduced by at least ~20-fold in eight mutants (Table S1). This plating defect was confirmed by titration of LL5 in full plate assays, in which only four mutants showed an EOP reduction of ~20-fold or greater. In order to confirm the plating phenotype in a clean genetic background, the kanamycin resistance cassettes from these Keio mutants were transduced by P1 into the parental *E. coli* strain BW25113. Markers could be transduced from all four Keio mutants into the parental strain, and three showed a similar plating defect as the corresponding Keio mutant, indicating the phenotype was linked to the disrupted locus (Table S1). One mutant, *waaQ*, showed a ~25-fold reduction in EOP in the Keio mutant but its P1 transductant exhibited only a very mild EOP defect of 0.3, despite having the *waaQ* deletion confirmed in the transductant by PCR and sequencing. This suggests an abnormality or additional defect in the original *waaQ* Keio mutant; this mutant was not examined further.

The same approach was applied to confirm the phenotypes of the Keio mutants identified from the screens against phage LL12 (Table S2). Spot titer assays showed that the EOP of phage LL12 was reduced in only

three of the nine initially identified Keio mutants. This plating defect could be replicated via full plate plaque assay in all three mutants. Only one of these mutants could be P1 transduced into the parental strain BW25113 and same plating phenotype was observed in the P1 transductant as in the Keio mutant (Table S2). The other two Keio mutants were resistant to P1 infection and could not be transduced.

Based on the genes identified in these initial screens, additional mutants from the odd- and even-numbered Keio sets were subjected to targeted re-screening by directly determining the phage EOP by the spot method. For both phages, genes involved in LPS biosynthesis (*waaP* for LL5, and *waaP, waaG* and *gmhA* for LL12, Tables S1, S2) were identified and confirmed, but these genes represented only parts of the known biosynthetic pathway. Additional Keio mutants in *gmhA*, *waaE*, *waaC*, *waaF*, *waaY*, *waaI*, and *waaB* were obtained and confirmed by PCR and sequencing of the mutant locus. A *tolC* mutant was obtained and tested based on the similarity between LL5 and phage TLS, which is known to use TolC as a receptor. Strong EOP defects ($< 10^{-7}$) were identified in the *gmhA*, *waaE*, *waaC*, *waaF,* and *tolC* mutants against LL5, and in *waaE*, *waaC* and *waaF* in LL12 (an EOP defect in *gmhA* against LL12 was already identified in the initial screen) (Table 2).

Discovery rates from gene knockout libraries

From the initial 37 "hits" identified in the untargeted screen, it was established that three *E. coli* genes were needed each for of phage LL5 and LL12 propagation (Tables S1, S2), which gives a false positive gene discovery rate of ~86% for both phages in the initial screen. It was noted that many genes predicted to play roles in phage propagation were not "hit" in the initial screen, so targeted screens were performed against additional mutants. Ultimately, eight genes affecting LL5 propagation and six genes affecting LL12 propagation were confirmed (Table 2), which translates to a false-negative gene discovery rate of at least 62% for LL5 and 50% for LL12 in the initial untargeted screen. In this process, it was discovered that in several instances the mutant genes were still intact in either the odd- or even-numbered sets, and in a few cases genes were still intact in both sets. These findings highlight the potential utility of using multiple

independently-generated mutant libraries when conducting large forward genetic screens as described here. Gene discovery rates are likely to be higher for biological pathways comprised of multiple genes, as this increases the probability that at least some genes in the pathway will be detected in initial screens. This highlights that the results from high-throughput forward genetic screens should be interpreted only after rigorous confirmation of the mutant genotypes and their phenotypes.

Table S1. Results of initial (untargeted) screening and targeted re-screening of phage LL5 against the Keio *E. coli* knockout collection. In the initial screen, all mutants yielding a positive result were screened a second time against both independently-generated gene knockouts present in the Keio collection, denoted as the representatives from the even- and odd-numbered plate sets. Mutants with a positive result from either set were then tested for their efficiency of plating (EOP) by both spot titer and full-plate titration methods. Mutants exhibiting a significant EOP defect (less than ~0.05, highlighted green) in full-plate titers were used for further study. The presence of the appropriate gene deletion was confirmed by PCR and sequencing. One knockout from each even/odd pair was selected for P1 transduction of the kan-marked deletion into the parental *E. coli* strain BW25113 background, re-tested for EOP defects and complemented in trans. Selected gene knockouts from the collection that were not identified in the initial screen were targeted for re-screening (bottom panel). Mutants were cultured from the Keio collection, the presence of the appropriate gene deletion confirmed by PCR, and the EOP determined in the knockout and its complemented counterpart. Blank cells denote that data was not collected, usually because the desired EOP defects were not observed.

Initial screen of Keio collection

Gene deletion	Results of first screen	Results of second screen		EOP (spot titer)		EOP (plate titer)		Keio mutant used for further work	Deletion confirmed by PCR	Transducible by P1*vir*	EOP (plate titer) in P1 transductant	EOP (plate titer) in complemented strain
		Odd	Even	Odd	Even	Odd	Even					
nuoM	+	+	+	0.06	0.04	0.3	0.3					
yehQ	+	-	-									
ycdB	+	+	-	0.4	0.2							
ydcR	+	+	-	1	1							
ygcN	+	-	-									
rof	+	-	-									
crcB	+	-	-									
ydjO	+	-	-									
holD	+	-	-									
aroC	+	-	-									
ydgL	+	-	-									
idi	+	-	-									
waaP	+	+	+	< 8E-8	1.0	< 7.5E-8	1.1	Odd	Confirmed	Yes	< 7.5E-8	2.2
waaG	+	+	-	1.1	0.3				Confirmed			
waaF	+	+	+	0.3	0.03	1.3	0.3					
waaQ	+	+	+	0.01	0.4	0.04	0.04	Odd	Confirmed	Yes	0.3	
rfaH	+	-	+	0.003	0.01	0.6	0.2					
waaY	+	-	-									
secB	+	+	+	0.01	0.03	0.05	0.02	Odd	Confirmed	Yes	0.06	0.2
ppiB	+	+	-	0.003	0.3	0.04	0.4	Odd	Confirmed	Yes	0.09	1.5
ylaC	+	+	-	0.07	0.03	0.4	0.8					
**Total passed**	**21**	**10**	**6**	**7**	**5**	**4**	**2**				**3**	

Table S1. Continued. Targeted re-screening. Based on the results of the initial screening, individual mutants were confirmed for gene deletions and obtained from other sources as necessary. The genes *waaC, waaF, and tolC* were found to be intact in the copy of the Keio collection used for initial screening.

Gene deletion	Deletion confirmed by PCR	Keio mutant used for further work	EOP (spot titer)	EOP (plate titer)	EOP (plate titer) in complemented strain (average ± S.D.)
gmhA	Confirmed	Even	< 8E-8	< 7.5E-8	0.8 ± 0.2
waaE	Confirmed	Even	< 8E-8	< 7.5E-8	1.0 ± 0.5
waaC	Confirmed	CGSC	< 2E-8	< 5.3E-7	0.7 ± 0.3
waaF	Confirmed	Dharmacon, Inc.	< 3E-7	< 7.9E-7	0.7 ± 0.3
waaY	Confirmed	Odd	1.4		
tolC	Confirmed	CGSC	< 3E-7	< 5.3E-7	0.4 ± 0.3

Table S2. Results of initial (untargeted) screening and targeted re-screening of phage LL12 against the Keio *E. coli* knockout collection. In the initial screen, all mutants yielding a positive result were screened a second time against both independently-generated gene knockouts present in the Keio collection, denoted as the representatives from the even- and odd-numbered plate sets. Mutants with a positive result from either set were then tested for their efficiency of plating (EOP) by both spot titer and full-plate titration methods. Mutants exhibiting a significant EOP defect (less than ~0.05, highlighted green) in full-plate titers were used for further study. The presence of the appropriate gene deletion was confirmed by PCR and sequencing. One knockout from each even/odd pair was selected for P1 transduction of the *kan*-marked deletion into the parental *E. coli* strain BW25113 background, re-tested for EOP defects and complemented *in trans*. Selected gene knockouts from the collection that were not identified in the initial screen were targeted for re-screening (bottom panel). Mutants were cultured from the Keio collection, the presence of the appropriate gene deletion confirmed by PCR, and the EOP determined in the knockout and its complemented counterpart. Blank cells denote that data was not collected, usually because the desired EOP defects were not observed.

Initial screen of Keio collection

Gene deletion	Results of first screen	Results of second screen		EOP (spot titer)		EOP (plate titer)		Keio mutant used for further work	Deletion confirmed by PCR	Transducible by P1*vir*	EOP (plate titer) in P1 transductant	EOP (plate titer) in complemented strain
		Odd	Even	Odd	Even	Odd	Even					
pflC	+	-	-									
cusB	+	-	-									
ompR	+	+	-	0.5	0.5							
envZ	+	-	-									
ompC	+	+	+	0.3	0.3							
yncJ	+	-	-									
ycbL	+	-	-									
yqjB	+	nt	nt	1.9	1.1							
yaaW	+	-	-									
yggT	+	+	+	0.1	0.1							
waaP	+	+	+	0.06	1.1	0.04	1.2	Odd	Yes	Yes	0.02	1.0
waaD	+	-	-									
waaG	+	+	-	7E-06	1.0	5.1E-06	1.3	Odd	Yes	No		1.1
waaF	+	-	-									
rfaH	+	+	-	0.4	0.8							
waaY	+	+	+	0.3	0.2							
rffE	+	+	-	0.2	0.1							
gmhA	+	-	+	0.8	< 4E-9	1.5	< 4.4E-9	Even	Yes	No		1.1
trmC	+	-	-									
ygbF	+	-	-									
etp	+	-	-									
**Total passed**	**21**	**8**	**5**	**2**	**1**	**2**	**1**				**1**	

Table S2. Continued. Targeted re-screening. Based on the results of the initial screening, individual mutants were confirmed for gene deletions and obtained from other sources as necessary. The genes *waaC* and *waaF* were found to be intact in the copy of the Keio collection used for initial screening.

Gene deletion	Deletion confirmed by PCR	Keio mutant used for further work	EOP (spot titer)	EOP (plate titer)	EOP (plate titer) in complemented strain (Average ± S.D.)
waaE	Confirmed	Even	< 4E-9	< 4.4E-9	1.1 ± 0.5
waaI	Confirmed	Odd	0.6		
waaB	Confirmed	Even	0.4		
waaC	Confirmed	CGSC	< 7E-8	< 5.1E-9	0.9 ± 0.4
waaF	Confirmed	Dharmacon, Inc.	< 8E-8	< 6.5E-9	1.5 ± 0.5
waaY	Confirmed	Odd	0.1		

Table S3. OD_{550} values for parental BW25113 and isogenic mutants that appeared as "hits" in the initial screen are presented below. The OD value reported is an average of four readings from different spots of a well of a 96-well plate and S.D. represents the standard deviation. Upper panel shows the OD value of bacterial culture in the screen against phage LL5. Lower panel shows the OD values of the bacterial culture in the screen against phage LL12. The OD values in the presence of phage are indicated by "+" sign followed by the name of the test phage.

	Average	S.D.
BW25113	0.40	0.01
BW25113 + LL5	0.06	0.01
Δ*waaP*	0.45	0.04
Δ*waaP* + LL5	0.39	0.03
Δ*secB*	0.65	0.05
Δ*secB* + LL5	0.49	0.05
Δ*ppiB*	0.42	0.06
Δ*ppiB* + LL5	0.39	0.08

	Average	S.D.
BW25113	0.38	0.02
BW25113 + LL12	0.05	0.01
Δ*waaP*	0.45	0.04
Δ*waaP* + LL12	0.38	0.06
Δ*gmhA*	0.39	0.02
Δ*gmhA* + LL12	0.41	0.02
Δ*waaG*	0.32	0.06
Δ*waaG* + LL12	0.32	0.07

Table S4: Homologs of putative LL12 tail fibers identified in related phage genomes.

LL12 Protein	Related phage containing tail fiber homolog	NCBI Genome Accession #	NCBI Protein Accession #	BLASTp E-value (vs. nr)	Dice similarity coefficient
**CPT_LL12_027**	rV5	NC_011041.1	YP_002003530.1	0	0.99
	APCEc02	KR698074.1	AKO61946.1	0	0.98
	phi92	NC_023693.1	YP_009012483.1	7.00E-23	0.08
	phi92	NC_023693.1	YP_009012482.1	2.00E-22	0.07
**CPT_LL12_029**	APCEc02	KR698074.1	AKO61944.1	0	0.99
	rV5	NC_011041.1	YP_002003532.1	0	0.99
	rV5	NC_011041.1	YP_002003535.1	2.00E-90	0.44
	APCEc02	KR698074.1	AKO61941.1	2.00E-90	0.44
	phi92	NC_023693.1	YP_009012479.1	6.00E-18	0.25
**CPT_LL12_032**	APCEc02	KR698074.1	AKO61941.1	0	0.99
	rV5	NC_011041.1	YP_002003535.1	0	0.99
	APCEc02	KR698074.1	AKO61944.1	6.00E-91	0.44
	rV5	NC_011041.1	YP_002003532.1	1.00E-88	0.44
	phi92	NC_023693.1	YP_009012479.1	9.00E-29	0.30
**CPT_LL12_033**	rV5	NC_011041.1	YP_002003536.1	0	1.00
	APCEc02	KR698074.1	AKO61940.1	0	1.00
	phi92	NC_023693.1	YP_009012474.1	0	0.41
**CPT_LL12_036**	APCEc02	KR698074.1	AKO61937.1	0	0.99
	rV5	NC_011041.1	YP_002003539.1	0	0.86
**CPT_LL12_041**	APCEc02	KR698074.1	AKO61932.1	0	0.96
	rV5	NC_011041.1	YP_002003543.1	0	0.70
	rV5	NC_011041.1	YP_002003545.1	3.00E-15	0.04
	APCEc02	KR698074.1	AKO61930.1	3.00E-15	0.04
	phi92	NC_023693.1	YP_009012483.1	3.00E-07	0.02
**CPT_LL12_042**	rV5	NC_011041.1	YP_002003544.1	0	0.99
	APCEc02	KR698074.1	AKO61931.1	0	0.99
	phi92	NC_023693.1	YP_009012473.1	2.00E-173	0.36

References

1. Baba, T., et al., *Construction of Escherichia coli K-12 in-frame, single-gene knockout mutants: the Keio collection.* Mol Syst Biol, 2006. **2**: p. 2006 0008.
